# Morphological and Rheological Guided Design for the Microencapsulation Process of *Lactobacillus paracasei CBA L74* in Calcium Alginate Microspheres

**DOI:** 10.3389/fbioe.2021.660691

**Published:** 2021-05-28

**Authors:** Concetta Di Natale, Elena Lagreca, Valeria Panzetta, Marianna Gallo, Francesca Passannanti, Michele Vitale, Sabato Fusco, Raffaele Vecchione, Roberto Nigro, Paolo Netti

**Affiliations:** ^1^Istituto Italiano di Tecnologia, IIT@CRIB, Largo Barsanti e Matteucci, Naples, Italy; ^2^Centro di Ricerca Interdipartimentale sui Biomateriali CRIB, University of Naples Federico II, Naples, Italy; ^3^Department of Chemical, Materials and Production Engineering, University of Naples Federico II, Naples, Italy; ^4^Department of Engineering, University of Rome Niccolò Cusano, Rome, Italy; ^5^Innovation & Technology Provider (ITP S.r.l.), Naples, Italy; ^6^Dipartimento di Medicina e Scienze della Salute “Vincenzo Tiberio”, Università del Molise, Campobasso, Italy

**Keywords:** microencapsulation, calcium alginate microsphere, multiple-particle tracking, probiotics, drug delivery

## Abstract

The intestinal microbiota is a real ecosystem composed of several bacterial species and a very huge amount of strains that through their metabolic activities play a crucial role in the development and performance of the immune system and other functions. Microbiota modulation by probiotics establishes a new era into the pharmaceutical and healthcare market. Probiotics play, in fact, an important role in helping and sustaining human health, but in order to produce benefits, their viability must be preserved throughout the production process up to consumption, and in addition, their bioactivity required to be safeguarded while passing through the gastrointestinal tract. In this frame, encouraging results come from encapsulation strategies that have proven to be very promising in protecting bacteria and their viability. However, specific effort has to be dedicated to the design optimization of the encapsulation process and, in particular, to the processing parameters that affect capsules microstructure. Herein, focusing on calcium alginate microspheres, after a preliminary selection of their processing conditions based on size distribution, we implemented a micro-rheological analysis, by using the multiple-particle tracking technique, to correlate the inner microstructure to the selected process conditions and to the viability of the *Lactobacillus paracasei CBA L74*. It was assessed that the explored levels of cross-linking, although changing the microorganism constriction, did not affect its viability. The obtained results confirm how this technology is a promising and a valid strategy to protect the microorganism viability and ensure its stability during the production process.

## Introduction

Microbiota plays a key role in the development of the immune system being its interaction with immune cells decisive for human health from early childhood (Yu et al., 2018). Its composition is very specific for every individual and seems to be strongly affected by dynamic changes and different dietary patterns and/or environmental conditions of the intestine (Salonen et al., 2014). Microbiota modulation by using probiotics constitutes a valuable strategy for the development of nutritional or pharmaceutical tools for healthcare (Ianiro et al., 2014; Sehrawat et al., 2020). Probiotics are usually defined as live microbial food ingredients able to provide beneficial effects on humans, including serum cholesterol level control, balance of intestinal microflora, enhancement of immunity defense, decrease in lactose intolerance, or anticariogenic activity (Lin, 2003). Anyway, these advantageous effects are linked to the concentration of probiotics reaching the intestine that should be at least of 10^6^ CFU/ml^6^. This implies that microorganisms, being taken orally, must be resistant to the passage through the gastrointestinal (GI) tract, surviving the action of gastric and bile juices (Scheinbach, 1998; Zoghi et al., 2019). In addition to the problem of probiotics’ survival in the passage through the GI tract, several studies have also shown low viability of probiotics bacteria in functional foods (Zoghi et al., 2019). These observations indicate the necessity to introduce a protective carrier, which can safely reach the intestine and provide the necessary concentrations for metabolic activities. Up to now, several methods have been performed to enhance probiotic viability, such as selection of strains tolerant to bile and acids or appropriate packaging materials, including protective compounds or oxygen scavengers (Sarkar, 2010). Among them, encapsulation has been reported to be the most useful method to protect probiotics from harmful environmental factors, such as high acidity and low pH levels, bile salts, and oxidation conditions (Scheinbach, 1998). This technology is used to “package” microorganisms cells in miniaturized capsules able to release it at controlled rates (Chávarri et al., 2010). Various polysaccharides as alginate, chitosan, or gellan gum have been employed to encapsulate probiotics (Tripathi and Giri, 2014); in particular, alginate is the most used thanks to its non-toxic nature, bioavailability, biocompatibility, low cost, and easy preparation as ionotropic gelation beads (George et al., 2019; Martãu et al., 2019). Specifically, alginate has been widely used as capsules materials to protect probiotic during the GI transit, and the stability of alginate beads has already been tested (Hansen et al., 2002; Ding and Shah, 2007; Cook et al., 2012; Holkem et al., 2016). However, even recently, there have been some efforts to further enhance the degree of protection of bacterial cells in the gastric conditions by microencapsulating them into alginate-dairy bases microcapsules or by using chitosan or poly-L-lysine-coated microspheres (MPs) (Martín et al., 2015; Yeung et al., 2016; Prasanna and Charalampopoulos, 2018).

Very importantly, the role of processing parameters should be thoroughly investigated for comprehensive understanding of how they influence microcapsule formation and microstructure and to overcome some of the limitations observed for alginate or other materials. To this purpose, we propose here the development of sodium alginate MPs with potential probiotic action *via* the water-in-oil emulsion technique and with inner microstructure properties that can be highly controlled by varying cross-linking agent (CaCl_2_) concentration and/or cross-linking time. In particular, after a preliminary selection of the processing conditions based on the analysis of size distribution, we adopted a micro-rheological analysis for an in-depth understanding on how processing parameters can affect inner microstructure, thus the probiotic viability and potentially release kinetics. To this aim, we implemented the multiple-particle tracking (MPT) technique to study the MPs rheology at different processing conditions. Indeed, MPT evaluates the diffusion of fluorescent probes embedded in a viscoelastic sample by studying their Brownian motion, directly related to the network’s mechanical properties, therefore to the cross-linkage degree (Moschakis, 2013). Then, we evaluated the post-production viability of microencapsulated *Lactobacillus paracasei CBA L74* at minimum and maximum cross-linking conditions. This microorganism is not able to withstand an acidic environment; therefore, encapsulation could be a good tool to ensure its protection. Its activity was assessed in both conditions meaning that the levels of constriction, induced by the polymer matrix associated to different cross-linkage levels, were not critical for the probiotics. Consequently, the entire selected cross-linking range is usable to tune alginate material degradation with consequent impact on the gastro-protection properties and on the kinetic release of the encapsulated compound that one may modulate depending on the GI compartment to be reached and treated. Moreover, as compared with classic mineral and paraffinic oils, which possess toxicity characteristics, a greener vegetable oil, namely, soybean oil, has been used as an outer emulsion phase.

## Materials and Methods

### Materials

The following materials were used: alginic acid sodium salt from brown algae (W201502; Sigma-Aldrich), calcium carbonate anhydrous, free-flowing, Redi-Dri^TM^ (CaCO_3,_ 795445, ACS reagent, ≥99%), soybean oil, dietary source of long-chain triglycerides and other lipids (S7381; Sigma-Aldrich), SPAN^®^ 80 (viscosity 1,000–2,000 mPa at 20°C; Sigma-Aldrich), acetic acid glacial (401406, ACS reagent; CARLO ERBA), calcium chloride dihydrate (CaCl_2_, ACS Reagent, ≥99%; Sigma-Aldrich), 200 nm yellow-green fluorescent (505–515), carboxylate-modified polystyrene nanoparticles (NPs) (Invitrogen Nanoprobes), *L. paracasei CBA L74* (Heinz Italia S.p.A., Latina, Italy), 20 g/L Bacto Yeast Extract (BD Biosciences, Milan, Italy), 0.5 g/L MgSO_4_ (Sigma-Aldrich, Milan, Italy), 50 g/L glucose (Sigma-Aldrich), and 0.5 g/L citric acid (Sigma-Aldrich).

### Methods

#### Microorganisms and Culture Conditions

*Lactobacillus paracasei CBA L74* is a Gram-positive homo-fermentative, facultative anaerobic bacteria for which a potential probiotic activity has been demonstrated by previous studies (Sarno et al., 2014; Gallo et al., 2019; Labruna et al., 2019). The strain was stored at −20°C and revitalized in 10 ml of an animal free broth (20 g/L Bacto Yeast Extract, 0.5 g/L MgSO_4_, 50 g/L glucose, 0.5 g/L citric acid) by incubation at 37°C. After 24 h, the suspension was centrifuged (1,600 rpm, 10 min), the supernatant discharged, and the pellet re-suspended in 10 ml of 2% w/v alginate.

#### Alginate MPs Preparation

Microspheres were prepared through the single emulsion water-in-oil technique by using CaCO_3_ as cross-linking agents. Particularly, the water phase was obtained by homogenization of 10 ml of 2% (w/v) alginate with 0.5 ml of CaCO_3_ with a concentration of 0.5 M by Ultra-Turrax (IKA T25 Digital) for 2 min at 3,000 rpm. This water phase was added drop by drop to 50 ml of the oil phase (soybean oil) with 500 μl of SPAN^®^ 80 and stirred at 200 rpm (Heidolph RZR 2102-BR 10) for 15 min. Then, a solution of 40 μl of acetic acid glacial and 10 ml of soybean oil was added to the W/O emulsion in order to obtain a pH variation able to promote the CaCO_3_ dissociation that allowed the first step of Ca^2+^-mediated cross-linking.

This first cross-linking phase was followed by a second one with the addition of several concentrations of CaCl_2_ (0.05, 0.1, 0.2 M) at different cross-linking times (5, 10, 15, 30, and 60 min). Based on these parameters, 15 different production formulations have been obtained and characterized (Table 1).

**TABLE 1:** Formulation tested in this study.

Formulation	(CaCl_2_) M	Cross-linking time (min)
F1	0.05	5
F2	0.05	10
F3	0.05	15
F4	0.05	30
F5	0.05	60
F6	0.1	5
F7	0.1	10
F8	0.1	15
F9	0.1	30
F10	0.1	60
F11	0.2	5
F12	0.2	10
F13	0.2	15
F14	0.2	30
F15	0.2	60

To allow MPs collection, these final emulsions were treated with 10% (v/v) TWEEN^®^ 20 to promote the separation between the two phases. The particles were washed with TWEEN^®^ 20 using a centrifuge at 25,000 rpm for 5 min at 4°C (SL16R Centrifuge; Thermo Scientific, United States) to remove soybean oil residues and to avoid aggregation phenomena during particle collection. To obtain the production yields of each formulation, MPs suspensions were filtered and then lyophilized overnight (−50°C, 0.73 hPa, Heto PowerDry PL6000 Freeze Dryer; Thermo Electron Corp., United States). The production yield was obtained by dividing the weight of lyophilized MPs with respect to the initial weight of polymer used for the preparation.

$$\%\text{yield}=\frac{g\,\text{lyophilizedMPs}}{g\,\text{alginate}}$$

**FIGURE 1:**
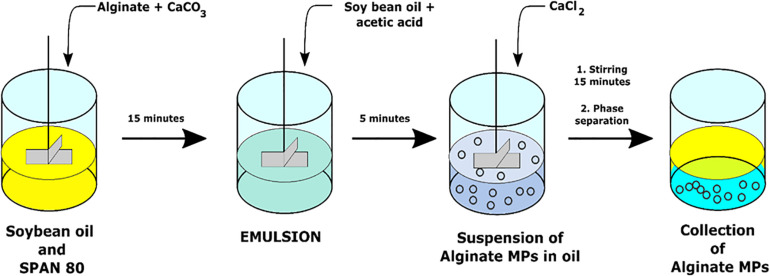
Schematic representation of alginate MS production.

The same preparation procedure was also used for MPs encapsulated *L. paracasei CBA L74* or fluorescent NPs (200 nm; Invitrogen Nanoprobes). In particular, NPs were encapsulated into alginate MPs by adding 33 μl of 1% solution of fluorescent NPs into 10 ml of 2% (w/v) alginate and 0.5 ml of 0.5 M CaCO_3_ before the homogenization step, whereas CBA L74-loaded-MPs were prepared by dissolving bacterial strain into 2% (w/v) alginate solution previously sterilized. All chemical reactions for MP synthesis are schematically represented in Figure 1.

### MP Characterization

#### Dimensional and Morphological Characterization: Optical Microscopy and Static Light Scattering

Each batch of alginate MPs was morphologically and dimensionally characterized by optical microscopy (OM) using an Inverted Microscope OLYMPUS IX73 magnified 40× by an oil objective (Di Natale et al., 2020, 2021). Moreover, the precise size of MPs was evaluated by static light scattering (LD) (Mastersizer 2000; Malvern Instruments, Malvern, United Kingdom) of 0.4 mg/ml alginate-MP suspension in TWEEN^®^ 20 (Celetti et al., 2016; Di Natale et al., 2018; Battisti et al., 2019; Jamaledin et al., 2020). Together with the average diameter (d50), for each size distribution, the SPAN value has also been evaluated, which is the distribution width calculated as:

$$\text{SPAN}=\left(\frac{\text{d}\,90-\text{d}\,10}{\text{d}\,50}\right)$$

where d90 is the particle diameter at which 90% of the particles is smaller than this value, whereas d10 is the diameter at which 10% of the particles is smaller than this value.

### Multiple-Particle Tracking

The role of cross-linking agent concentration [CaCl_2_ (0.05, 0.1, and 0.2 M)] on the radial distribution of MPs network mesh-size was evaluated through the MPT technique. Videos of fluorescent 200 nm polystyrene-FITC-NPs embedded in alginate MPs (50 MPs for each sample) were acquired in time-lapse for a total time of 10 s at 10 frames per second (fps), using an inverted fluorescence microscope (Olympus IX81; Olympus), equipped with a 60× water immersion objective (high numerical aperture, N. A. 1.3) and a Hamamatsu ORCA-Flash 2.8 CMOS camera (Hamamatsu). The trajectories of fluorescent NPs were obtained by using our self-developed MATLAB 7 code. By this routine, each particle position was determined by intensity measurements of different areas and localized by each area’s centroid; afterward, it was compared frame by frame to produce the trajectory of each particle, based on the principle that the two closest positions in successive frames belong to the same particle (proximity principle). Then, mean square displacements (MSDs) curves were calculated from NPs trajectories using equation (a) and fitted by equation (b):

$$a)\text{MSD}=\frac{1}{N}\sum {}^{N}{}_{t=1}<[R_{i}\left(t\right)-R_{i}\left(0\right)]{}^{2}>$$

$$b)\text{MSD}=2nDt{}^{\alpha }$$

where *n* is the dimension of the system (2 in this case), *D* is the diffusion coefficient (μ*m*^2^/*s*), *t* is the time (s), and α is a non-dimensional parameter, which describes the way of motion (free diffusion α = 1, sub-diffusive α < 1, or super-diffusive α > 1). Curve fitting with a coefficient of determination (*R*^2^) less than 0.5 was discarded from the analysis and considered not reliable from a statistical point of view. The radial diffusion map of investigated MPs was determined by correlating the diffusion coefficient *D* to the starting position of each tracked particle. In particular, the MP centroid position and radium were calculated by image analysis using the freeware NIH software (ImageJ 1.37c). From NPs trajectories, the distances between the initial position of NPs and alginate MPs centroids were obtained and normalized by MPs radium (*r*/*R*). The normalized distance was divided into 10 sections to allow the statistical analysis. For each section, the mean value of *D* was plotted as a function of normalized distance. All data were compared with a non-polarized alginate solution.

### Microbiological Assay

The viability of *L. paracasei CBA L74* after encapsulation was evaluated by MRS Agar assay (Oxoid, United Kingdom). After serial dilutions, substrate was spread on Petri dishes of MRS agar and incubated at 37°C for 72 h at the end of which it is possible to count the colonies formed on each plate. Plate inseminations were carried out pre- and post-microencapsulation. Before the microencapsulation process, an insemination was carried out using an alginate sample in which the bacterial strain was dispersed.

The entrapped probiotics were instead evaluated dissolving the MPs into 1% w/w sodium citrate solution at pH 6.

## Results and Discussion

### MP Production and Characterization

**FIGURE 2:**
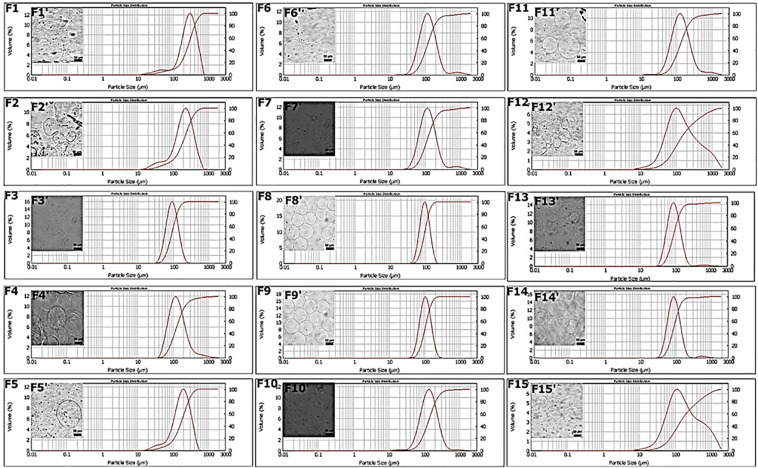
Chemical–physical **(F1–F2)** and morphological characterization **(F1′–F2′)** of microspheres.

Microspheres were produced by the single emulsion method as reported in the Materials and Methods section. By combining two different processing parameters, cross-linking concentration and time of production, 15 different production formulations were obtained and characterized. The objective of these first experiments was to carry out a prior screening of the MPs produced at various cross-linking concentrations and at different times, based on the dimensional parameter calculated with two different techniques, such as Mastersizer and OM. In particular, the target diameter was set at 100 μm since larger particle diameters could alter the quality of the final product (Zuidam and Shimoni, 2010; Lavelli et al., 2014), and the value of the SPAN parameter, which indicates the width of the diameter distribution curve, was calculated as described in the “Materials and Methods” section. These parameters were evaluated for all formulations and reported in Table 2. The obtained results showed that the F1 and F2 formulations displayed non-symmetrical distributions ranging from 10 to 700 μm and high value of SPAN between 1.6 and 1.8 (Figures 2F1–F2). This behavior can be explained by the presence of pronounced aggregation phenomena between MPs (Figures 2F1′–F2′). Better results were obtained for the F3 formulation, which showed an average diameter of 93.10 ± 0.12 μm and a SPAN value of 0.9 (Figures 2F3–F3′). On the contrary, increasing the cross-linking time (F4 and F5) curves with a very wide distribution were obtained together with average diameters larger than 100 μm (Figures 2F4–F5, F4′–F5′). The distribution curves obtained with 0.1 M cross-linking agent showed a constant trend; indeed, for all formulations, the mean diameter was near the target value of 100 μm. In particular, F8 and F9 (Figures 2F8–F9) revealed high symmetrical distribution with SPAN values less than 1, and their mono-dispersion was also confirmed by OM (Figures 2F8′–F9′). As to the formulation F10, even if the distribution was symmetrical, the SPAN value was 1.3 indicating the beginning of aggregation phenomena also confirmed by optical images (Figures 2F10–F10′). The widest and least symmetrical distributions were obtained for F6 and F7 (SPAN of 1.7 and 1.8, respectively), corresponding to cross-linking times of 5 and 10 min (Figures 2F6–F7, F6′–F7′), maybe not enough to provide sufficient cross-linking. Similar results were obtained for formulations with 0.2 M CaCl_2_; they showed highly variable distribution curves according to the cross-linking time. Particularly, the curve of formulation F11, relating to the time of 5 min, showed the presence of a peak relative to particles with diameters greater than 1,000 μm and a SPAN value of 1.4 (Figure 2F11). These values are due to aggregation phenomena between MPs (Figure 2F11′). The same considerations were for the F12, in which the distribution curve is non-uniform with a SPAN value of 3.7 and an average diameter of 116 μm (Figures 2F12–F12′). For formulations F13 and F14, relating to the cross-linking times of 15 and 30 min, no differences were observed in terms of peaks of the distributions (Figures 2F13–F14). The average diameter settles around the target value of 100 μm, and the SPAN values are slightly greater than 1. However, it is possible to note how, even if these formulations showed the most homogeneous distribution curves (Figures 2F13–F14), a second peak related to particles of about 500 μm was found, confirming the occurrence of aggregation phenomena (Figures 2F13′–F14′). The last formulation tested, F15, displayed a fewer uniform distribution with a SPAN value of 4.2 despite the average diameter recorded was equal to 105.84 ± 2.94 μm (Figures 2F15–F15′).

**TABLE 2:** Values of d50 and SPAN for all the studied formulations, *n* = 3.

Formulation	(CaCl_2_) M	Cross-linking time (min)	d50 (μ m)	SPAN
F1	0.05	5	297.87 ± 2.72	1.6
F2	0.05	10	191. 95 ± 3.48	1.8
F3	0.05	15	93.10 ± 0.12	0.9
F4	0.05	30	128.08 ± 3.69	1.6
F5	0.05	60	156.88 ± 2.20	1.5
F6	0.1	5	97.71 ± 1.18	1.7
F7	0.1	10	106.25 ± 0.48	1.8
F8	0.1	15	95.10 ± 1.37	0.8
F9	0.1	30	104.27 ± 2.23	0.9
F10	0.1	60	105.73 ± 1.00	1.3
F11	0.2	5	86.74 ± 0.25	1.4
F12	0.2	10	116.51 ± 0.87	3.7
F13	0.2	15	90.72 ± 0.89	1.1
F14	0.2	30	88.54 ± 0.15	1.1
F15	0.2	60	105.84 ± 2.94	4.2

Therefore, from the analysis of particle diameters, we concluded that time affects the quality of the particle distribution below and above certain values. For times below 15 min especially for the minimum concentration of cross-linking agent equal to 0.05 M, there is aggregation due to low cross-linkage. For all the concentrations, the minimum time to have good quality particles with SPAN close to or below 0.1 is 15 min. Remarkably, in all the cases, the maximum cross-linking time of 60 min always promoted some aggregation, which was particularly evident for the maximum cross-linker concentration of 0.2 M. For the minimum cross-linker concentration (0.05 M), the increase of the SPAN value was registered already at 30 min. SPAN increase at prolonged cross-linking times indicates some aggregation most probably due to a cross reticulation between particles, whereas for short cross-linking times especially for lower cross-linker concentrations, processing conditions are not sufficient to stabilize structurally the particles that can undergo coalescence as well as differentiated *swelling* or *shrinkage* phenomena (Oliveira and Mano, 2011). An analysis on production yields was also performed. As shown in Supplementary Table 1, at each cross-linking concentration, the yield was improved by increasing the cross-linking time. A similar trend was obtained by setting the cross-linking time, in this case, the yield enhanced as the concentration increased. This behavior can be justified by the diffusion kinetics of Ca^2+^ cations that become faster when both parameters grow. The best result was obtained using the cross-linking time of 30 min where a yield of 96% was achieved at 0.2 M (Supplementary Table 1). The lowest yield (27%) was instead obtained for the formulation with the lowest parameters: 0.05 M and 15 min (Supplementary Table 1). In this case, the combination of the two parameters is not sufficient to ensure that the cross-linking phenomenon is homogeneous for all the droplets of alginate dispersed in the oil phase within the emulsion. Moreover, the 0.05 M concentration was able to reach only 39% of the production yield at 30 min, which instead for the concentration of 0.1 M was achieved already at 15 min (Supplementary Table 1). One future aim will be to optimize process conditions in order to improve the production yield for the selected cross-linking conditions.

### Microencapsulation of *L. paracasei CBA L74*

Based on MPT data in which the condition of minimum cross-linking guarantees greater mobility to the encapsulated component, whereas the condition of maximum cross-linking immobilizes the encapsulated component in a dense polymeric network, we decided to carry out the microencapsulation tests of *L. paracasei CBA L74* in the critical conditions of minimum cross-linkage (F3: 0.05 M CaCl_2_, 15 min) and maximum cross-linkage (F14: 0.2 M CaCl_2_, 30 min). These two extreme conditions among the formulations gave us the best results in terms of SPAN. A less dense cross-linking should guarantee greater mobility to the microorganism, whereas a complete cross-linking should immobilize the microorganism in the polymerized alginic acid.

Viability tests confirmed that in both cases, minimum and maximum cross-linking conditions, the encapsulated microorganism remained viable, maintaining the initial bacterial load unaltered. In detail, as shown in Table 3, no significant differences were found when the data obtained in the post-encapsulation phase were compared with those of the initial microbial load related to bacterial strain dispersed in alginate solution at time *t*_0_. That means that at least in terms of strain viability, the explored range of processing conditions is viable for further investigation.

**TABLE 3:** Evaluation of *Lactobacillus paracasei CBA L74* viability before and after encapsulation processes.

	Strain (CFU ml^–1^)	*t*_0_ (CFU ml^–1^)	Post-encaps (CFU ml^–1^)
**F3**	1.98 × 10^8^	5.03 × 10^8^	1.57 × 10^8^
**F14**	1.98 × 10^8^	1.33 × 10^8^	1.54 × 10^8^

*The measurement of the number of colonies reported was in CFU ml^–1^ (Colony Forming Units); *n* = 3.*

### MPT

After a first screening based on the size of the average diameter, MPs were investigated in terms of microstructure by implementing an innovative technique based on MPT. MPT is a micro-rheological technique able to investigate the rheological properties of a polymeric network by studying the mobility of NPs embedded within the polymer matrix. Such mobility is connected to the MPs cross-linking degree, which determines MP functionality. First of all, the cross-linking degree is linked to mechanical stresses to which the encapsulated components are subjected, which in the case of *L. paracasei CBA L74*, could lead to a possible decrease in the bacterial load. Anyway, at least in the explored range, this circumstance was excluded by the viability test performed at the extremes of such range. Moreover, the cross-linking degree is connected to the degree of protection against the surrounding environment and to the release kinetics of the encapsulated components. First, we checked by MPT if a complete and uniform cross-linking within the MPs was obtained.

**FIGURE 3:**
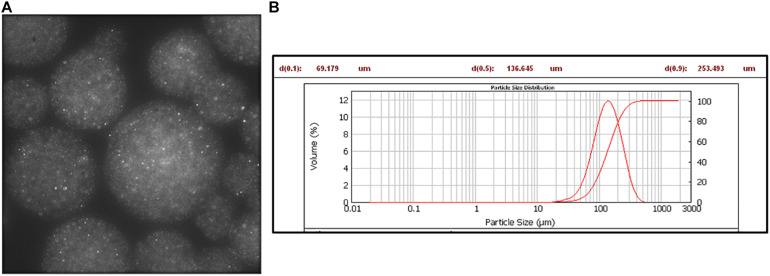
Fluorescent NPs encapsulated in alginate microspheres. **(A)** Fluorescence image: λ_exc_ 488 nm, λ_emiss_ 520–600 nm. **(B)** Mastersizer analysis.

**FIGURE 4:**
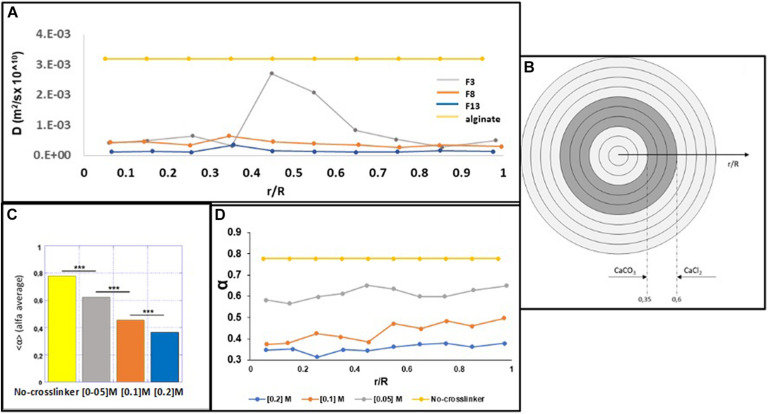
MPT microsphere analysis. **(A)** The *D* coefficient was correlated to the normalized distance along the radius of the microspheres for all formulations. **(B)** Internal and external gelation mechanisms for the concentration of 0.05 M CaCl_2_ and the non-polymerization area of the bare alginate. **(C)** Average values of α for the bare alginate and F3, F8, and F13 formulations. **(D)** α Values are plotted as a function of the normalized distance along the radius from the center of the microspheres.

In particular, by using 200 nm fluorescent NPs embedded into the alginate MPs, we calculated the diffusivity coefficients along the normalized radius of the MPs (Figures 3A,B), as described in the “Materials and Methods” section. Figure 4A shows the results obtained by plotting the diffusion coefficient (*D*) as a function of the normalized distance along the MPs radius. Comparing the samples at 15 min (F3, F8, and F13), the coefficient *D* was almost constant along the radius of the MPs for both F8 and F13 formulations and lower for the 0.2 M concentration (F13), whereas for the 0.05 M concentration (F3), the diffusion coefficient did not show a constant trend with a peak in correspondence to the normalized radius value equal to 0.45 close to the diffusivity of the alginate solution (∼3.2 × 10^–3^ μm^2^/s). We interpreted this behavior as related to an uncompleted outside-in CaCl_2_ polymerization process during the MP fabrication. In particular, we supposed that the polymerization process was stopped when the diffusion of the divalent Ca^2^^+^ cations covered about 40% of the radius (Figure 4B). In other words, when a concentration of 0.05 M was used for 15 min of cross-linking time, the polymerization front due to the external gelation mechanism did not advance along the entire MP radius. To complete the polymerization and obtain a uniform cross-linking within the MPs, a cross-linking time of 30 min (F4) was necessary (Supplementary Figure 1). Conversely, for the 0.1 and 0.2 M cross-linker concentrations, a time of 15 min was sufficient to guarantee a uniform and complete cross-linking within MPs. Starting from these observations, we compared the three formulations obtained at 15 min (F3, F8, and F13) analyzing their motion regime. The parameter (α) was obtained by fitting the MSD of NPs with a power-law equation as described in the “Materials and Methods” section and was used to obtain information on the mode of motion of NPs encapsulated within MPs. In detail, α equal to one identifies a purely diffusive regime, α less than one identifies a sub-diffusive regime, and α greater than one identifies a super-diffusive regime.

In Figure 4C, the average values of α for the formulations F3, F8, and F13, compared with the value obtained by analyzing the starting solution of 2% uncured alginate (v/w), are shown. The identified motion regimes were all sub-diffusive, and as expected, α parameter decreases when the cross-linking concentration rises up. This is made evident in Figure 4D, where α is plotted as a function of the normalized distance along the radius from the MP center. For each concentration of tested cross-linker, the NPs mobility remains almost unchanged (the percentage variation is ≃1%) from the center to the outer MP section and decreases as the cross-linker concentration increases. Figures 4A,C shows that the parameters α and *D* present an inverse correlation with the cross-linker concentration (both decreasing with increasing the cross-linker concentration), suggesting that the mesh-size of the polymer network is lowering, posing a steric hindrance for NPs mobility. Furthermore, the power law dependence of the MSD on the time lag is a signature of mechanical behavior of the polymer network (Fusco et al., 2015; Panzetta et al., 2017); thus, the reduction of *D* can be considered accompanied by a stiffening process of the alginate MPs when the cross-linker concentration increases. Importantly, the possibility to control the mechanical properties of the MPs microstructure can be used to finely tune their degradation and then the kinetic release of the encapsulated compound.

Thanks to the MPT analysis, we can understand the impact of the processing parameters (cross-linker concentration and cross-linking time) toward inner microstructure parameters (*D* and α), which, upon future *in vitro* digestion tests, can help in the optimal design of the MPs.

## Conclusion

This study presents a simple method for the encapsulation of the probiotic *L. paracasei CBA L74* in sodium alginate MPs by the water-in-oil emulsion technique. The optimization of the formulation parameters was obtained by varying cross-linking agent concentrations and cross-linking times and by replacing mineral and paraffinic oils with a greener and safer vegetable oil. Then, once shortlisted, the parameters ranges, an MPT based micro-rheological analysis was performed within the MPs in order to understand the relation between processing parameters and inner microstructures, which in turn can affect probiotic viability and its release. Post-production viability of microencapsulated *L. paracasei CBA L74* was assessed at minimum and maximum cross-linking conditions meaning that the entire selected cross-linkage range is viable to tune MP microstructure. Additionally, we could understand the impact of the processing parameter on MP properties (ex. *D* and α), which can help in the optimal design of the system upon future *in vitro* digestion tests. The latter tests will indeed provide useful feedback on the MP degradation and, therefore, on the gastro-protection and release kinetic properties, which will be correlated to the MPs properties. In this way, a fine tuning of the processing parameters will be theoretically performed and then experimentally assessed. The final aim will be to ensure the viability of the microorganism and, at the same time, its release into the colon and place the bases for application in the industrial field, particularly in the food industry. Subsequent studies will concern the development of a functional food with beneficial properties for the intestinal microbiota.

## Data Availability Statement

The original contributions presented in the study are included in the article/Supplementary Material, further inquiries can be directed to the corresponding author/s.

## Author Contributions

CD and RV prepared the draft and the final version of the manuscript. CD, EL, and MV performed the MP synthesis and morphological characterization. VP, MV, and SF achieved the MPT analysis. VP and SF also revised the original draft. MG and FP performed the biological assays and revised the original draft. RV, RN, and PN conceived the research and revised the original draft. All authors contributed to the article and approved the submitted version.

## Conflict of Interest

The authors declare that the research was conducted in the absence of any commercial or financial relationships that could be construed as a potential conflict of interest.

## Supplementary Material

Supplementary Figure 1 *D* coefficient of F3 and F4 formulations along the radius (0–1r).

## References

[B1] Battisti M. Vecchione R. Casale C. Pennacchio F. A. Lettera V. Jamaledin R. (2019). Non-invasive production of multi-compartmental biodegradable polymer microneedles for controlled intradermal drug release of labile molecules. *Front. Bioeng. Biotechnol.* 7:296. 10.3389/fbioe.2019.00296 31781550 PMC6856554

[B2] Celetti G. Di Natale C. Causa F. Battista E. Netti P. A. (2016). Functionalized poly (ethylene glycol) diacrylate microgels by microfluidics: in situ peptide encapsulation for in serum selective protein detection. *Colloids Surf. B Biointerfaces* 145 21–29. 10.1016/j.colsurfb.2016.04.036 27137799

[B3] Chávarri M. Marañón I. Ares R. Ibáñez F. C. Marzo F. del Carmen Villarán M. (2010). Microencapsulation of a probiotic and prebiotic in alginate-chitosan capsules improves survival in simulated gastro-intestinal conditions. *Int. J. Food Microbiol.* 142 185–189. 10.1016/j.ijfoodmicro.2010.06.022 20659775

[B4] Cook M. T. Tzortzis G. Charalampopoulos D. Khutoryanskiy V. V. (2012). Microencapsulation of probiotics for gastrointestinal delivery. *J. Control. Release* 162 56–67. 10.1016/j.jconrel.2012.06.003 22698940

[B5] Di Natale C. Celetti G. Scognamiglio P. L. Cosenza C. Battista E. Causa F. (2018). Molecularly endowed hydrogel with an in silico-assisted screened peptide for highly sensitive small molecule harvesting. *Chem. Commun.* 54 10088–10091. 10.1039/c8cc04943b 30116812

[B6] Di Natale C. De Rosa D. Profeta M. Jamaledin R. Attanasio A. Lagreca E. (2021). Design of biodegradable bi-compartmental microneedles for the stabilization and the controlled release of the labile molecule collagenase for skin healthcare. *J. Mater. Chem. B* 9 392–403. 10.1039/d0tb02279a 33283828

[B7] Di Natale C. Onesto V. Lagreca E. Vecchione R. Netti P. A. (2020). Tunable release of curcumin with an in silico-supported approach from mixtures of highly porous PLGA microparticles. *Materials* 13:1807. 10.3390/ma13081807 32290458 PMC7215757

[B8] Ding W. Shah N. (2007). Acid, bile, and heat tolerance of free and microencapsulated probiotic bacteria. *J. Food Sci.* 72 M446–M450. 18034741 10.1111/j.1750-3841.2007.00565.x

[B9] Fusco S. Panzetta V. Embrione V. Netti P. A. (2015). Crosstalk between focal adhesions and material mechanical properties governs cell mechanics and functions. *Acta Biomater.* 23 63–71. 10.1016/j.actbio.2015.05.008 26004223

[B10] Gallo M. Nigro F. Passannanti F. Nanayakkara M. Lania G. Parisi F. (2019). Effect of pH control during rice fermentation in preventing a gliadin P31-43 entrance in epithelial cells. *Int. J. Food Sci. Nutrit.* 70 950–958. 10.1080/09637486.2019.1599827 30969137

[B11] George A. Shah P. A. Shrivastav P. S. (2019). Natural biodegradable polymers based nano-formulations for drug delivery: a review. *Int. J. Pharm.* 561 244–264. 10.1016/j.ijpharm.2019.03.011 30851391

[B12] Hansen L. T. Allan-Wojtas P. Jin Y.-L. Paulson A. (2002). Survival of Ca-alginate microencapsulated *Bifidobacterium* spp. in milk and simulated gastrointestinal conditions. *Food Microbiol.* 19 35–45. 10.1006/fmic.2001.0452

[B13] Holkem A. T. Raddatz G. C. Nunes G. L. Cichoski A. J. Jacob-Lopes E. Grosso C. R. F. (2016). Development and characterization of alginate microcapsules containing Bifidobacterium BB-12 produced by emulsification/internal gelation followed by freeze drying. *LWT Food Sci. Technol.* 71 302–308. 10.1016/j.lwt.2016.04.012

[B14] Ianiro G. Bibbo S. Gasbarrini A. Cammarota G. (2014). Therapeutic modulation of gut microbiota: current clinical applications and future perspectives. *Curr. Drug Targets* 15 762–770. 10.2174/1389450115666140606111402 24909808

[B15] Jamaledin R. Sartorius R. Di Natale C. Vecchione R. De Berardinis P. Netti P. A. (2020). Recombinant filamentous bacteriophages encapsulated in biodegradable polymeric microparticles for stimulation of innate and adaptive immune responses. *Microorganisms* 8:650. 10.3390/microorganisms8050650 32365728 PMC7285279

[B16] Labruna G. Nanayakkara M. Pagliuca C. Nunziato M. Iaffaldano L. D’Argenio V. (2019). Celiac disease−associated *Neisseria flavescens* decreases mitochondrial respiration in CaCo−2 epithelial cells: impact of *Lactobacillus paracasei* CBA L74 on bacterial−induced cellular imbalance. *Cell. Microbiol.* 21:e13035. 10.1111/cmi.13035 PMC6618323 31042331

[B17] Lavelli V. Harsha P. S. Torri L. Zeppa G. (2014). Use of winemaking by-products as an ingredient for tomato puree: the effect of particle size on product quality. *Food Chem.* 152 162–168. 10.1016/j.foodchem.2013.11.103 24444921

[B18] Lin D. C. (2003). Probiotics as functional foods. *Nutrit. Clin. Pract.* 18 497–506. 16215085 10.1177/0115426503018006497

[B19] Martãu G. A. Mihai M. Vodnar D. C. (2019). The use of chitosan, alginate, and pectin in the biomedical and food sector—biocompatibility, bioadhesiveness, and biodegradability. *Polymers* 11:1837. 10.3390/polym11111837 31717269 PMC6918388

[B20] Martín M. J. Lara-Villoslada F. Ruiz M. A. Morales M. E. (2015). Microencapsulation of bacteria: a review of different technologies and their impact on the probiotic effects. *Innov. Food Sci. Emerg. Technol.* 27 15–25. 10.1016/j.ifset.2014.09.010

[B21] Moschakis T. (2013). Microrheology and particle tracking in food gels and emulsions. *Curr. Opin. Colloid Interface Sci.* 18 311–323. 10.1016/j.cocis.2013.04.011

[B22] Oliveira M. B. Mano J. F. (2011). Polymer−based microparticles in tissue engineering and regenerative medicine. *Biotechnol. Progr.* 27 897–912. 10.1002/btpr.618 21584949

[B23] Panzetta V. Musella I. Rapa I. Volante M. Netti P. A. Fusco S. (2017). Mechanical phenotyping of cells and extracellular matrix as grade and stage markers of lung tumor tissues. *Acta Biomater.* 57 334–341. 10.1016/j.actbio.2017.05.002 28483699

[B24] Prasanna P. Charalampopoulos D. (2018). Encapsulation of *Bifidobacterium longum* in alginate-dairy matrices and survival in simulated gastrointestinal conditions, refrigeration, cow milk and goat milk. *Food Biosci.* 21 72–79. 10.1016/j.fbio.2017.12.002

[B25] Salonen A. Lahti L. Salojärvi J. Holtrop G. Korpela K. Duncan S. H. (2014). Impact of diet and individual variation on intestinal microbiota composition and fermentation products in obese men. *ISME J.* 8 2218–2230. 10.1038/ismej.2014.63 24763370 PMC4992075

[B26] Sarkar S. (2010). Approaches for enhancing the viability of probiotics: a review. *Br. Food J.* 112 329–349. 10.1108/00070701011034376

[B27] Sarno M. Lania G. Cuomo M. Nigro F. Passannanti F. Budelli A. (2014). *Lactobacillus paracasei* CBA L74 interferes with gliadin peptides entrance in Caco-2 cells. *Int. J. Food Sci. Nutrit.* 65 953–959. 10.3109/09637486.2014.940283 25030417

[B28] Scheinbach S. (1998). Probiotics: functionality and commercial status. *Biotechnol. Adv.* 16 581–608. 10.1016/s0734-9750(98)00002-0 14538145

[B29] Sehrawat N. Yadav M. Singh M. Kumar V. Sharma V. R. Sharma A. K. (2020). Probiotics in microbiome ecological balance providing a therapeutic window against cancer. *Semin. Cancer Biol.* 70 24–36. 10.1016/j.semcancer.2020.06.009 32574811

[B30] Tripathi M. K. Giri S. K. (2014). Probiotic functional foods: survival of probiotics during processing and storage. *J. Funct. Foods* 9 225–241. 10.1016/j.jff.2014.04.030

[B31] Yeung T. W. Üçok E. F. Tiani K. A. McClements D. J. Sela D. A. (2016). Microencapsulation in alginate and chitosan microgels to enhance viability of *Bifidobacterium longum* for oral delivery. *Front. Microbiol.* 7:494. 10.3389/fmicb.2016.00494 27148184 PMC4835488

[B32] Yu Q. Jia A. Li Y. Bi Y. Liu G. (2018). Microbiota regulate the development and function of the immune cells. *Int. Rev. Immunol.* 37 79–89. 10.1080/08830185.2018.1429428 29425062

[B33] Zoghi A. Khosravi-Darani K. Sohrabvandi S. Attar H. Alavi S. A. (2019). Survival of probiotics in synbiotic apple juice during refrigeration and subsequent exposure to simulated gastro-intestinal conditions. *Iran. J. Chem. Chem. Eng. (IJCCE)* 38 159–170.

[B34] Zuidam N. J. Shimoni E. (2010). “Overview of microencapsulates for use in food products or processes and methods to make them,” in *Encapsulation Technologies for Active Food Ingredients and Food Processing*, eds Zuidam N. Nedovic V. (New York, NY: Springer), 3–29. 10.1007/978-1-4419-1008-0_2

