# Supplementary material for: Morphological and Rheological Guided Design for the Microencapsulation Process of Lactobacillus paracasei CBA L74 in Calcium Alginate Microspheres
Source: Front Bioeng Biotechnol. 2021 May 28;9:660691. doi: 10.3389/fbioe.2021.660691 (PMC8193927; doi:10.3389/fbioe.2021.660691)
Supplement: Supplementary Figure 1 — D coefficient of F3 and F4 formulations along the radius (0–1r). [file Data_Sheet_1.docx]

Supporting Information

**Morphological and rheological guided design for the microencapsulation process of Lactobacillus paracasei CBA L74 in calcium alginate microspheres**

**Concetta Di Natale,^1,2^ Elena La Greca,^1^ Valeria Panzetta,^1,2,3^ Marianna Gallo^3,4,5^, Francesca Passannanti^5^, Michele Vitale^3^, Sabato Fusco^2,6^, Raffaele Vecchione^1,*,^ Roberto Nigro^3,*^ and Paolo Netti^1,2,3^**

^1 Istituto Italiano di Tecnologia, IIT@CRIB, Largo Barsanti e Matteucci, 53 80125 Napoli, Italy.^

^2 Centro di Ricerca Interdipartimentale sui Biomateriali CRIB, Università di Napoli Federico II, Piazzale Tecchio, 80 80125 Napoli, Italy.^

^3 Department of Chemical, Materials and Production Engineering, University of Naples Federico II, Piazzale Tecchio 80, 80125, Naples, Italy.^

^4 University of Rome Niccolò Cusano, Engineering Department, Via Don Carlo Gnocchi, 3, 00166, Rome, Italy.^

^5 Innovation & Technology Provider (ITP S.r.l.), Via Bisignano a Chiaia 68, 80121 Naples, Italy.^

^6 Dipartimento di Medicina e Scienze della Salute “Vincenzo Tiberio”, Università del Molise, Via Francesco De Sanctis 1, Campobasso, 86100, Italy.^

*** Correspondence:**Raffaele.vecchione@iit.it

Roberto.nigro@unina.it

**Figure S1:** D coefficient of F3 and F4 formulations along the radius (0-1r).

**Table S1: Production yields (*%)***

| **[CaCl_2_] M** | **15 min** | **30 min** |
| --- | --- | --- |
| **0.05** | 27% (F3) | 39% (F4) |
| **0.1** | 40% (F8) | 60% (F9) |
| **0.2** | 68% (F13) | 96% (F14) |
